# Expression of a Stilbene Synthase Gene from the *Vitis labrusca* x *Vitis vinifera* L. Hybrid Increases the Resistance of Transgenic *Nicotiana tabacum* L. Plants to *Erwinia carotovora*

**DOI:** 10.3390/plants11060770

**Published:** 2022-03-14

**Authors:** Elena B. Rukavtsova, Valeriya V. Alekseeva, Sergey V. Tarlachkov, Natalia S. Zakharchenko, Alexander A. Ermoshin, Svetlana A. Zimnitskaya, Alexey K. Surin, Elena Y. Gorbunova, Viatcheslav N. Azev, Sergey S. Sheshnitsan, Konstantin A. Shestibratov, Yaroslav I. Buryanov

**Affiliations:** 1 Department of Biomolecular Chemistry, Branch of Shemyakin-Ovchinnikov Institute of Bioorganic Chemistry, Russian Academy of Sciences, 142290 Pushchino, Moscow Region, Russia; ruk@bibch.ru (E.B.R.); zachar@bibch.ru (N.S.Z.); 2 Department of Plant Molecular Biology and Biotechnology, Branch of Shemyakin-Ovchinnikov Institute of Bioorganic Chemistry, Russian Academy of Sciences, 142290 Pushchino, Moscow Region, Russia; lera@bibch.ru (V.V.A.); buryanov@bibch.ru (Y.I.B.); 3 Department of Biotechnology, Branch of Shemyakin-Ovchinnikov Institute of Bioorganic Chemistry, Russian Academy of Sciences, 142290 Pushchino, Moscow Region, Russia; sergey@tarlachkov.ru (S.V.T.); elena@bibch.ru (E.Y.G.); viatcheslav.azev@bibch.ru (V.N.A.); 4 All-Russian Collection of Microorganisms (VKM), G. K. Skryabin Institute of Biochemistry and Physiology of Microorganisms, Pushchino Scientific Center for Biological Research of the Russian Academy of Sciences, 142290 Pushchino, Moscow Region, Russia; 5 Department of Experimental Biology and Biotechnology, Institute of Natural Science and Mathematics, Ural Federal University, 620002 Ekaterinburg, Sverdlovsk Region, Russia; alexander.ermoshin@urfu.ru; 6 Department of Biology and Fundamental Medicine, Institute of Natural Science and Mathematics, Ural Federal University, 620002 Ekaterinburg, Sverdlovsk Region, Russia; s.a.zimnitskaya@urfu.ru; 7 Department of Biological Testing, Branch of Shemyakin-Ovchinnikov Institute of Bioorganic Chemistry, Russian Academy of Sciences, 142290 Pushchino, Moscow Region, Russia; alan@vega.protres.ru; 8 Laboratory of Bioinformatics and Proteome Investigations, Institute of Protein Research, Russian Academy of Sciences, 142290 Pushchino, Moscow Region, Russia; 9 State Research Center for Applied Microbiology and Biotechnology, 142279 Obolensk, Moscow Region, Russia; 10 Faculty of Forestry, Voronezh State University of Forestry and Technologies Named after G.F. Morozov, 8 Timiryazeva Str., 394087 Voronezh, Voronezh Region, Russia; sheshnitsan@gmail.com

**Keywords:** *Nicotiana tabacum* L., stilbene synthase, transgenic plants, pathogens, resveratrol, flavonoids, pollen

## Abstract

‘Isabel’ grape (*Vitis labrusca* x *V. vinifera* L. hybrid) is one of the main grape cultivars in Russia and some other countries for processing, due to its vigor, tolerance to the main fungal diseases, high yield and potential for sugar accumulation. The stilbene synthase gene *VlvSTS* was isolated from the hybrid grape cv. Isabel and cloned into a pSS plant transformation vector under the control of a constitutive 35S RNA double promoter of the cauliflower mosaic virus, CaMV 35SS. *VlvSTS*-gene containing transgenic tobacco lines were obtained and analyzed. For the first time plants expressing the *VlvSTS* gene were shown to have an enhanced resistance to the bacterial pathogen *Erwinia carotovora* subsp. *carotovora* B15. Transgenic plants were tested for resistance to a number of fungal pathogens. The plants were resistant to the grey mould fungus *Botrytis cinerea*, but not to the fungi *Fusarium oxysporum*, *F. sporotrichioides*, or *F. culmorum*. According to the results of a high performance liquid chromatography-mass spectrometry analysis, the amount of trans-resveratrol in leaves of transgenic plants with the highest expression of the *VlvSTS* gene was in a range from 150 to 170 μg/g of raw biomass. Change in the color and a decreased anthocyanin content in the flower corollas of transgenic plants were observed in transgenic lines with the highest expression of *VlvSTS*. A decrease in total flavonoid content was found in the flower petals but not the leaves of these tobacco lines. High expression of the *VlvSTS* gene influenced pollen development and seed productivity in transgenic plants. The size of pollen grains increased, while their total number per anther decreased. A decrease in the number of fertile pollen grains resulted in a decreased average weight of a seed boll in transgenic plants.

## 1. Introduction

Creation of pathogen-resistant plants relies on a number of strategies. One of them is to develop plants capable of synthesizing low-molecular antimicrobial compounds, phytoalexins, e.g., stilbenes. Stilbenes are found in plants of different families, but the best studied stilbene is resveratrol (3,5,4′-trihydroxystilbene) isolated from grape plants. Resveratrol synthesis in plant cells occurs via the phenylpropanoid pathway and is regulated by the enzyme stilbene synthase (EC 2.3.1.95). It was shown that transgenic plants expressing stilbene synthase genes may have a higher resistance to pathogens. The resistance of some tobacco lines expressing grape *Vst1* and *Vst2* genes to the fungus *B. cinerea* was first established by Hain et al., 1993 [1]. Resveratrol synthesis in transgenic plants did not always render them fully resistant to one or another pathogen. Transgenic rice plants with the grape stilbene synthase *Vst1* gene were partially resistant to the fungus *Pyricularia oryzae* [2]. Papaya plants transformed by the same gene were better, although not fully, protected against the pathogen *Phytophthora palmivora* compared with non-transformed control [3]. Other researchers used grape and pine stilbene synthase genes under their own promoters for wheat transformation [4]. It was shown that transgenic wheat resistance to different pathogens varied greatly: leaf damage symptoms decreased by 19–27% in plants infected with *Puccinia recondita*, and by 42–71% in plants infected with *Septoria nodorum*. Expression of *Pinus* stilbene synthase gene—which leads to the synthesis of pinosilvin rather than resveratrol—did not render wheat resistant to pathogens. Transgenic tomato plants with grape stilbene synthase genes were less susceptible to the fungus *Phytophthora infestans*, but lacked resistance to *B. cinerea* and *Alternaria solani* [5]. Although they synthesized stilbenes, transgenic kiwi plants were not resistant to *B. cinerea* pathogens [6]. Some studies observed almost complete plant resistance to pathogens [7,8]. The use of stilbene synthase genes from wild-type grapes *V. quinquangularis* and *V. amurensis* for *Arabidopsis* transformation yielded conflicting data on plant resistance to biotrophic and necrotrophic pathogens [9,10].

The stilbene synthase genes from *Vitaceae* are developmentally regulated and induced by infection of pathogens. The annotation of the near-homozygous PN40024 genotype of the *V. vinifera* cv. Pinot noir genome made it possible to identify up to 48 putative *VvSTS* gene sequences. At least 33 full length sequences encoding *VvSTS* genes were identified, which, based on predicted amino acid sequences, cluster in 3 principal groups A, B and C [11,12]. The existence of multiple *STS* genes in *V. vinifera* genome suggests a lead role of the *STS*-mediated pathways in the adaptation of this species to environmental challenges. 

To date, a rather large set of grape varieties resistant to biotic and abiotic factors, derived from interspecific crosses with *V. labrusca* L. genotypes, has been created. The *V. labrusca* L. grape is the most famous and widespread American grape species, which was the earliest of all American species introduced to Europe. The practical interest in *V. labrusca* L. lies primarily in the fact that as result of the associated evolution of this species with the main agents of fungal diseases (powdery and downy mildew, gray mold) and pests (phylloxera) common on the American continent, it has acquired resistance and can be a valuable donor in breeding work carried out by interspecific hybridization methods. Moreover, the *V. labrusca* L. species have high frost and drought resistance. ‘Isabel’ grape (*Vitis labrusca* x *V. vinifera* L. hybrid) is one of the main grape cultivars in Russia and some other countries for processing, due to its vigor, tolerance to the main fungal diseases, high yield and potential for sugar accumulation [13]. Previously, the dependence of the expression of genes of stilbene synthases of ‘Isabel’ grapes on infection with downey mildew *Plasmopara viticola* was shown [14]. 

The aim of our investigation was to isolate a *VlvSTS* stilbene synthase gene from the hybrid grape *V. labrusca* x *V. vinifera* L. and obtain transgenic tobacco plants overexpressing the gene to study their resistance to a number of bacterial and fungal pathogens, among them bacteria as *E. carotovora* and fungi as *B. cinerea, F. oxysporum*, *F. sporotrichioides*, *F. culmorum*. It should be noted that no earlier studies evaluated the resistance against *E. carotovora* in transgenic plants synthesizing resveratrol. Based on previous knowledge that overexpression of stilbene synthase gene can lead to male sterility in plants [15,16,17], we studied the effect of the *VlvSTS* gene expression on the synthesis of anthocyanins, flavonoids, as well as on pollen and seed development in transgenic plants.

## 2. Results

### 2.1. Cloning of the Stilbene Synthase Gene VlvSTS, Obtaining Tobacco Plants with the Gene and Performing Their Molecular Biological Analysis

**Figure 1:**
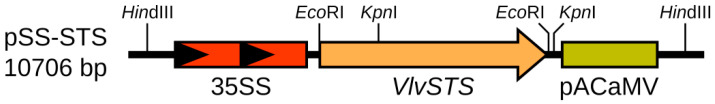
Schematic diagram of pSS-STS plasmid. CaMV35SS and pACaMV—promoter with two enhancer domains and polyadenylation signal of cauliflower mosaic virus 35S RNA; *VlvSTS—*stilbene synthase gene of hybrid grape *V. labrusca* x *V. vinifera* L.; *npt*II—gene of neomycin phosphotransferase II.

The stilbene synthase gene *VlvSTS* was isolated from leaves of the hybrid grape *V. labrusca* x *V. viniferaL*. cv. Isabel and cloned into a pSS plant binary vector under the double promoter of the cauliflower mosaic virus 35S RNA (CaMV 35SS) [18]. The nucleotide sequence of *VlvSTS* was identified, it was 98.6% identical to that the *V. vinifera* L. stilbene synthase gene *vinst1* (GenBank AB046375.1). In the hybrid grape cultivar ‘Isabel’ (*V. labrusca* x *V. vinifera* L.), the VlvSTS stilbene synthase amino acid sequence had histidine in position 58, whereas in the *V. vinifera* L. stilbene synthase the same position was occupied by arginine. A scheme of a pSS vector-based plasmid carrying the *VlvSTS* gene is presented in Figure 1. Several tobacco lines with the *VlvSTS* gene of grape stilbene synthase were obtained and studied. The presence of the *VlvSTS* and neomycin phosphotransferase *npt*II genes in the genome of the obtained plants was confirmed by PCR. For further studies, we selected 8 transgenic lines whose DNAs were shown to contain DNA fragments matching in size both with *VlvSTS* gene and the internal fragment of the *npt*II gene.

**Figure 2:**
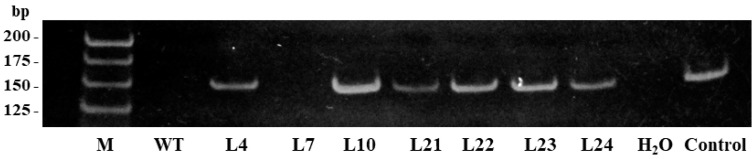
RT-PCR amplicons generated from cDNA of leaves of transgenic tobacco transformed with *VlvSTS* gene. M—HyperLadder 25 bp DNA molecular weight marker (Meridian Life Bioscience, Memphis, USA), WT—nontransformed tobacco, L4–L24—transgenic tobacco lines transformed with *VlvSTS* gene, H_2_O—water, Control—PCR-product of *VlvSTS* gene fragment (150 bp).

The expression of the *VlvSTS* gene in transgenic lines (TL) was first tested by semi-quantitative RT-PCR. Fragments of 150-bp corresponding to the *VlvSTS* transcript were identified in TL, whereas no such transcripts were detected in control lines (Figure 2). *VlvSTS* expression was not observed in line L7. 

**Figure 3:**
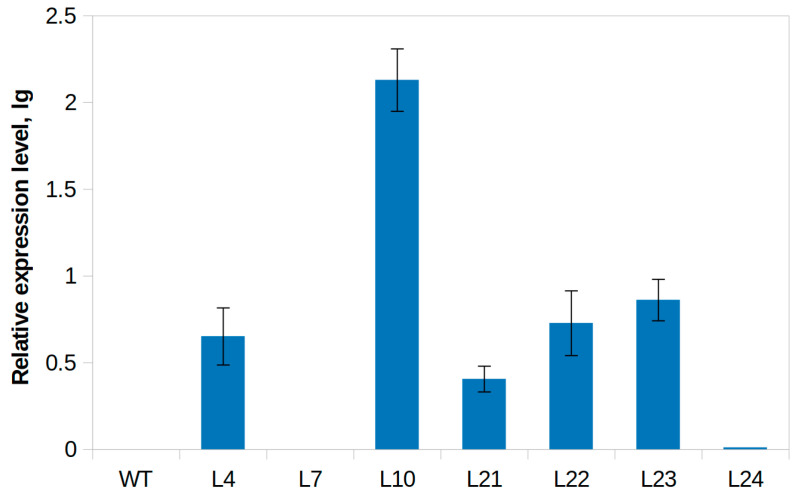
Expression level (in logarithmic scale) of the *VlvSTS* gene in the leaves of transgenic tobacco plants. WT—control tobacco, L4–L24 are transgenic tobacco lines with the *VlvSTS* gene. The gene of the elongation factor *EF1α* was used as reference, the mRNA sample with the lowest transcription level (line L24) was used as calibrator.

The relative expression level of the *VlvSTS* gene in the TL was determined using quantitative real-time RT-PCR (qPCR) (Figure 3). According to qPCR data, the expression level of the *VlvSTS* gene was minimum in L24 and maximum in the line L10, with the maximum interline difference in expression exceeding a hundred times. The absence of gene transcription in line L7 was confirmed by this analysis. Based on the results of the *VlvSTS* gene transcription analysis, seven transgenic tobacco lines were selected for further experiments.

### 2.2. Resistance of Transgenic Plants to Pathogens

**Figure 4:**
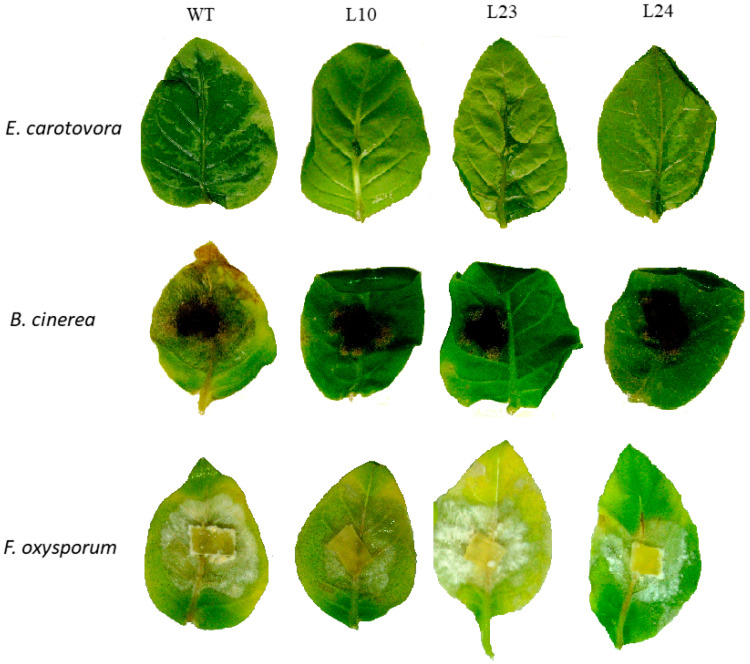
Leaf resistance in transgenic tobacco plants transformed with *VlvSTS* gene to pathogenic bacteria *E. carotovora,* fungi *B. cinerea* and *F. oxysporum*. WT—leaves of non-transformed plants, L10, L23, and L24—leaves of transgenic plants.

**Figure 5:**
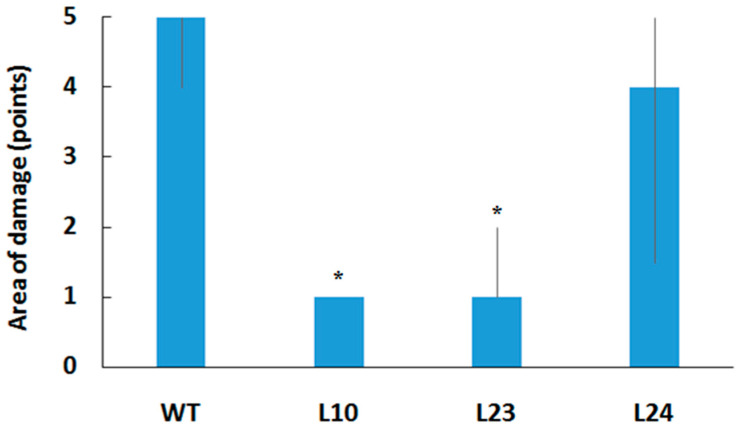
Resistance of leaves of tobacco plants transformed with *VlvSTS* gene to pathogenic bacteria *E. carotovora*. Extent of leaf damage 48 h post inoculation. Median, Q1 and Q3 are shown for the area of leaf damage scored from 0 to 5 points, 0 = no damage, 1 = 10–20% damage, 2 = 20–40%, 3 = 40–60%, 4 = 60–80%, 5 = 80–100%. * Statistically significant at Mann-Whitney test (*p* < 0.05).

The results of the biotests for plant leaf resistance against studied pathogens are shown in Figure 4. In all tests with *E. carotovora* bacteria, transgenic plants expressing the *VlvSTS* gene demonstrated a significantly higher resistance versus control plants (Figure 4). A few hours after being infected with *Erwinia*, leaves of the control plants showed the first signs of incipient tissue damage around the wound surface of the leaf central vein. On the second day, almost the entire leaf surface was affected by necrosis. The leaf involvement was significantly smaller in transgenic plants expressing the *VlvSTS* gene. In L10 and L23 plants, with the highest gene expression, up to 80% of leaf tissue remained healthy (Figure 5). We also tested the TL lines for resistance to the fungal pathogens *F. oxysporum*, *F. sporotrichioides*, *F. culmorum*, and *B. cinerea*. The extent of plant damage due to fungal pathogens was estimated 7–14 days after exposure. There was a significant reduction of disease symptoms after infection of TL leaves by *B. cinerea*, but not to *Fusarium* fungi (Figure 4).

### 2.3. Trans-Resveratrol Content Assay in Leaves of the Tobacco Transgenic Line with the Highest Expression of the VlvSTS Gene

**Figure 6:**
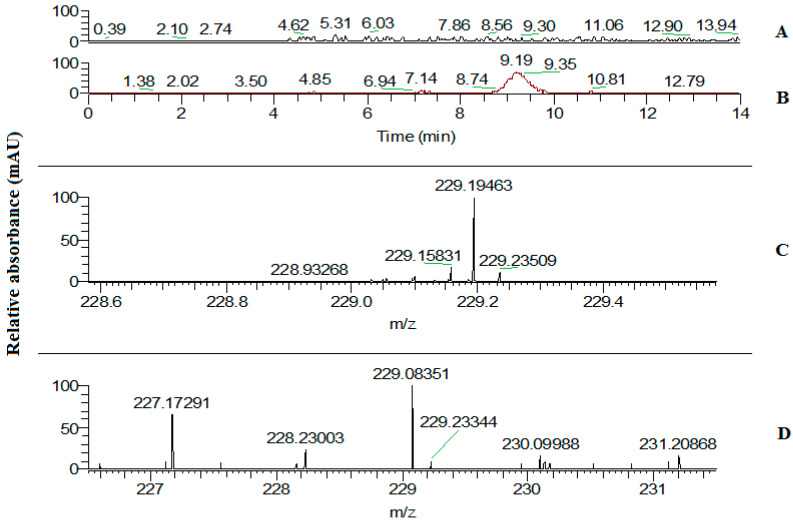
Mass chromatograms (**A**,**B**) and mass spectra (**C**,**D**) for control and experimental samples (extracts from leaves of control plants and transgenic L10 plants expressing the *VlvSTS* gene). The chromatograms show that there were no ions with the mass *m*/*z* 229.083 in the control sample (**A**), whereas the chromatographic peak of resveratrol was observed in the experimental sample (**B**). The lower part of the figure shows mass spectrum of ions formed from products eluted out of the column at 9.19 min. The control sample had no peak corresponding to the mass of 229.083 (**C**), while that was the major peak presented by the experimental sample (**D**).

Since the stilbene synthase gene is responsible for resveratrol synthesis in plants, we determined the content of this metabolite in the leaves of transgenic plants. This was accomplished by high performance liquid chromatography-mass spectrometry analysis of leaf extracts derived from plants of the L10 tobacco line with the highest expression of the *VlvSTS* gene (Figure 6). The amount of trans-resveratrol in the leaves of TL was in a range from 150 to 170 μg/g of raw biomass, which is comparable to the content of this metabolite in grape leaves, or to its content in grape skin upon stress conditions. According to other studies in transgenic plants expressing a stilbene synthase gene, trans-resveratrol and trans-piceid (resveratrol glucoside) levels range from 0.1 to 650 μg/g of crude biomass [8,19,20,21,22,23].

### 2.4. Anthocyanin Content in Flower Corollas in Transgenic Plants

**Figure 7:**
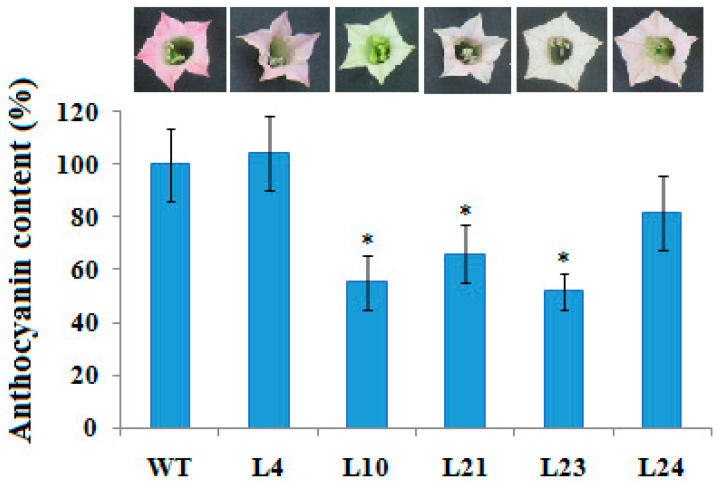
Decrease in the anthocyanin content in flower petals of transgenic tobacco plant lines transformed with the *VlvSTS* gene (%).The content of anthocyanins in control plants is taken as 100%. WT—non-transformed plant. L4, L10, L21, L23, L24—lines of transgenic plants. * Statistically significant at Mann-Whitney test (*p* < 0.05).

We studied the effect of the introduced gene on the reproductive properties of plants. Plants of all transgenic lines with the *VlvSTS* gene were grown in greenhouse for further analysis and seed production. Overexpression of the *VlvSTS* gene reduced the corolla pigmentation in transgenic plants. In tobacco plants of line L10, with high *VlvSTS* gene expression, the buds and immature flowers were white, and only maturing flowers got a slight pink pigmentation. In transgenic line L7 with a ‘silent’ *VlvSTS* gene, flowers did not differ from control. The total anthocyanin content in the flower corollas of transgenic plants decreased to 51.9% of that in control (Figure 7). This might have been the result of a competition for substrates between the two enzymes, stilbene and chalcone synthases [11].

### 2.5. Flavonoid Content in Flower Corollas and Leaves of Transgenic Plants

In tobacco lines with high expression of the *VlvSTS* gene (L10 and L23), the flower total flavonoid content lowered by 24–26% compared to non-transformed control. Yet, there was no decrease in leaf flavonoid content in these lines. The decreased flavonoid content in the flower organs could affect the reproductive properties of transgenic tobacco plants. 

### 2.6. The Effect of VlvSTS Gene Expression on Pollen and Seed Development in Transgenic Plants

**Figure 8:**
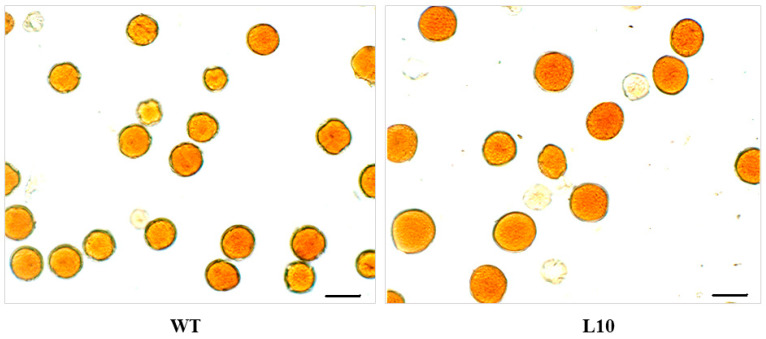
Pollen stained with acetocarmine. WT—nontransformed plant, L10—transgenic plant transformed with *VlvSTS* gene. Bar = 30 μm.

**Figure 9:**
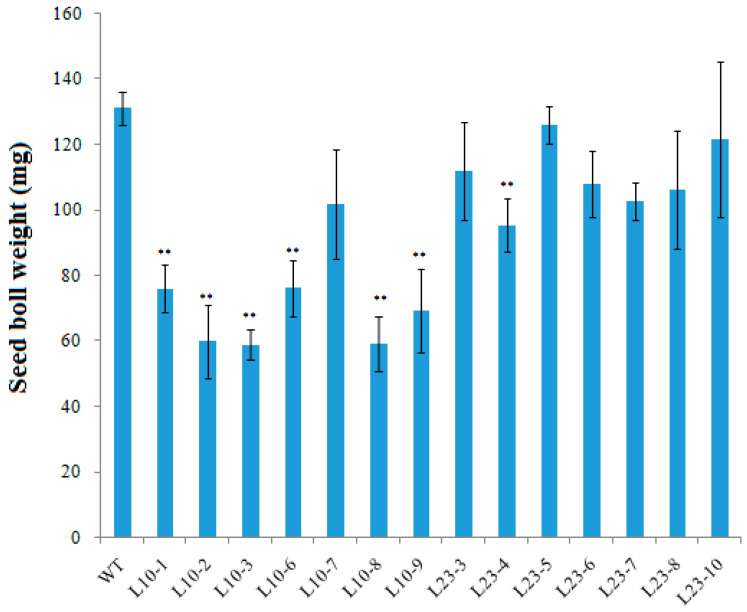
Average seed boll weight in transgenic tobacco plants with *VlvSTS* gene (mg). Seed boll weight in control plants was taken as 100%. WT—nontransformed plant; L10 and L23–T0 transgenic plant lines; L10-2, L10-6, L10-8, L10-9, L23-5, L23-6, L23-8, L23-9—T1 transgenic plant lines. ** Statistically significant at Mann-Whitney test (*p* < 0.01).

Expression of the *VlvSTS* gene in tobacco plants led to a significant increase in the pollen grain size, but with a tendency to a decrease in the total number of pollen grains per anther (Table 1, Figure 8). The number of fertile pollen grains also decreased. Line L10, with the highest expression of the *VlvSTS* gene, showed a significant fertility reduction, maximum increase in pollen grain volume and a significant change in pollen amount. These changes resulted in a decreased weight of a seed boll in the transgenic tobacco lines. The mean weight of a seed boll decreased to 46% of that in control (Figure 9).

## 3. Discussion

Stilbene synthase involvement in rendering plants resistant to various pathogens was shown by many authors [1,2,3,4,5,7,8,9,10]. It is related to the synthesis of various stilbenes in plants, and among them resveratrol, which is an antioxidant and phytoalexin. In this study, we analyzed transgenic tobacco plants transformed with a stilbene synthase gene *VlvSTS* from thehybrid grape *Vitis labrusca* x *Vitis vinifera* L. cv. Isabel under the control of the double promoter CaMV 35SS. The gene expression was various in different transgenic lines, as shown by real-time RT-PCR. For the first time it was shown that plants expressing the *VlvSTS* gene had enhanced resistance to the bacterial pathogen *E. carotovora* subsp. *carotovora* B15. The study evaluated three lines of transgenic plants with a high, medium, and low *VlvSTS* expression (lines L10, L23 and L24). Interestingly, resistance correlated with the expression level of the stilbene synthase gene. In plants with a higher gene expression (L10 and L23), the area of *Erwinia*-caused leaf damage was in a range from 0 to 40% respect to the control, while the leaves of line L24 were affected up to 60–100%. In control plants, the pathogen caused cell lysis over the entire leaf surface, while in transgenic lines, the lysis areas were significantly smaller, probably due to the synthesis of the phytoalexin resveratrol.

We determined the resistance of isolated leaves of the obtained plants to the fungal pathogens *F. oxysporum*, *F. sporotrichioides*, *F. culmorum*, and *B. cinerea*. We demonstrated the plant resistance to grey mould caused by *Botrytis cinerea*, which is consistent with previous studies [1,10]. Our transgenic plants, however, were susceptible to *Fusarium* infection. Fungi of the *Fusarium* genus are widespread soil fungi, some of which are serious pathogens causing rot and wilt of roots, stems and fruits [24]. Other authors showed an increased resistance of transgenic *Rehmannia glutinosa* plants expressing the peanut stilbene synthase gene *AhRS3* to the pathogen *F. oxysporum* infecting roots [8]. The hypothesis could be that pathogensof the *Fusarium* genus infect the leaves rather than the roots of transgenic plants, resveratrol synthesis will not be sufficient to protect against such pathogens.

Some studies showed that *Arabidopsis* plants transformed with wild grape stilbene synthase genes responded to a pathogen attack by activating genes responsible for the synthesis of signaling molecules such as salicylic and jasmonic acids (SA and JA, respectively) [9,10]. The SA participation in plant-pathogen interactions can be complex and ambiguous. It is thought that necrotrophic pathogens, such as *E. carotovora* and *B. cinerea*, induce the formation of SA to cause the local death of host cells, which is associated with release of nutrient substrate from them [25,26].

Such pathogens induce an oxidative burst and hypersensitivity reaction (HSR) in plants. Their virulence is directly related to the ROS content in the host tissues. In mutant plants unable to start HSR, the growth of these pathogens was suppressed, although it was activated upon artificial stimulation of HSR [25]. JA is involved in both resistance reactions and susceptibility of plant to the pathogens. The antagonism between SA and JA pathways can allow pathogens manipulate the defence reactions by the plant. It is not yet clear how the constitutive synthesis of the phytoalexin resveratrol in our transgenic plants can affect the regulation and activation of protective responses against various pathogens.

Our data show a decrease in the total flavonoid content in the flower petals but not in the leaves of plants upon expression of the *VlvSTS* gene. This is a very important observation given the multiple functions of flavonoids in a plant, from plant development regulation, pigmentation and protection from UV radiation to the many roles in protection against pathogens and signal transmission between plants and microorganisms [27,28]. The significant decrease in the flavonoid content, including anthocyanins, in the flower organs can affect the reproductive properties of transgenic tobacco plants, which is consistent with previous studies [15]. We were the first to show that transgenic tobacco plants carrying the *VlvSTS* gene had a significantly larger pollen grain size and a smaller number of pollen grains per anther. At the same time, the number of fertile pollen grains decreased, especially in the plant line with the highest expression of the *VlvSTS* gene. These changes resulted in a decreased weight of seed bolls in the transgenic tobacco lines. It was previously shown that overexpression of the stilbene synthase gene in tomatoes caused male sterility and parthenocarpy [17]. According to some other researchers, however, the stilbene synthase gene does not always affect pollen development. It probably depends both on the gene source and the type of promoter, as well as on the species of the transformed plant. For example, in apple plants with the grape stilbene synthase gene *Vst1* under its own promoter, the expression of the gene had no effect on pollen development [29].

It should be noted that the *VlvSTS* gene, which was shown to confer resistance against pathogenic microorganisms in the obtained transgenic plants, may be promising for use for plant biotechnology purporses. The obtained vector constructs with the stilbene synthase gene can be further used to study the gene effects in other plants of interest. A decrease in the flower flavonoid content in transgenic plants can influence their color and pollen development to the extent that plants may become male sterile. This property of the stilbene synthase gene may be useful for obtaining safer transgenic plants unable to cross pollinate with wild species.

## 4. Materials and Methods

### 4.1. Plants, Growth Conditions

The study used plants and seeds of the of tobacco *Nicotiana tabacum* L. cv. Samsun. The plants were grown in vitro in 0.5–1 L culture bottles, on phytohormone-free Murashige and Skoog medium (MS) [30] containing 7 g/L agar and 30 g/L sucrose (pH 5.8), at 24–26 °C, with a 16-h photoperiod, 2000 Lx light intensity, and a relative humidity of 65%. The rooted plants were transferred to a greenhouse to obtain seeds. The seeds were sterilized in 70% ethanol for 1 min, then in 2–3% sodium hypochlorite solution for 10 min, and washed 5 times in sterile distilled water for 10 min. The seeds were germinated on the agarized MS medium. The seeds of transgenic tobacco lines were selected in the presence of the antibiotic kanamycin sulfate (100 mg/L).

Plants of the the hybrid grape *Vitis labrusca* x *Vitis vinifera* L. cv. Isabel were grown indoors in a Biotron artificial climate chamber.

### 4.2. Strains of Pathogenic Microorganisms

We used a bacterial strain *E. carotovora*subsp. *carotovora* B15 obtained from the Horticulture Centre (Canada) and fungal pathogens *F. oxysporum*, *F. sporotrichioides*, *F. culmorum*, and *B. cinerea* obtained from the All-Russian Collection of Microorganisms of the Institute of Biochemistry and Physiology of Microorganisms of the Russian Academy of Sciences (http://www.vkm.ru). 

### 4.3. Designing of a pSS-STS Vector for Plant Transformation

The stilbene synthase gene *VlvSTS* was isolated from leaves of the ‘Isabel’ hybrid grape *Vitis labrusca* x *Vitis vinifera* L. and cloned into a pSS plant binary vector under the control of the double promoter of the cauliflower mosaic virus 35S RNA, CaMV 35SS [18]. To synthesize the gene, we developed oligonucleotide primers based on the *vinst1* grape gene (GenBank AB046375.1). The total RNA was isolated from grape leaves as described by Bekesiova et al. [31], with minor modifications. The isolated RNA was dissolved in RNase-free water (Evrogen, Moscow, Russia), and its concentration was measured on a Shimadzu UV-1800 spectrophotometer (Shimadzu, Kyoto, Japan). The RNase inhibitor Ribolock (Thermo Fisher Scientific, Vilnius, Lithuania) was added to the RNA and the resultant solution was kept at −70 °C. Part of the RNA was treated with DNase I (Thermo Fisher Scientific, Vilnius, Lithuania). Reverse transcription of the RNA template was carried out with the reverse transcriptase MMLV (Evrogen, Moscow, Russia). Oligo(dT) primer was used for cDNA synthesis. Polymerase chain reaction (PCR) was conducted on a cDNA template with the gene primers 5′-CGGAATTCATGGCTTCAGTTGAGG-3′ and 5′-CGGAATTCTTAATTTGTAACTGTAGGAACG-3′. A restriction site *Eco*RI included into the oligonucleotide primers was added at both ends of the *VlvSTS* gene. The PCR conditions were as follows: 5 min at 94 °C; 30 cycles: 30 s at 94 °C, 30 s at 61 °C, 2 min at 72 °C, followed by 7 min at 72 °C, on an MJ Mini Personal Thermal Cycler (Bio-Rad Laboratories, Singapore, Singapore).

The DNA of the *VlvSTS* gene synthesized by RT-PCR was treated with the *Eco*RI enzyme and joined with the pBluescriptII KS+ plasmid hydrolyzed at the same restriction site using the T4 DNA ligase (Thermo Fisher Scientific, Vilnius, Lithuania). *E. coli* TG2 cells were transformed with this mixture [32]. The nucleotide sequence of the gene was determined (Evrogen, Moscow, Russia). The nucleotide and amino acid sequence data are available in GenBank (Accession number OK626589.1). The plasmid DNA of the selected clones with the *VlvSTS* gene was hydrolyzed with the *Eco*RI restriction endonuclease, and the resulting DNA fragment was introduced into the pSS plant transformation vector [18] hydrolyzed with the *Eco*RI enzyme. The gene orientation relative to the CaMV 35SS promoter was determined by DNA hydrolysis at the *Kpn*I restriction site. The obtained plasmid pSS-STS was transferred to the *Agrobacterium tumefaciens* GV3101 (pMP90RK) strain [33] by direct transformation [34].

### 4.4. Plant Transformation

Tobacco plants were transformed by inoculating leaf discs with agrobacteria [35]. After the transformation, explants were transferred onto a selective MS medium containing 1 mg/L BAP, 0.1 mg/L NAA, 50 mg/L kanamycin sulfate, and 500 mg/L cefotaxime. After 3 weeks time, shoot regeneration was observed in the explants. Each experiment was carried out in 10–15 Petri dishes with 10 explants per dish. Tobacco shoots selected on a selective medium were checked by PCR analysis. Thus we obtained 8 lines of transgenic tobacco plants with the *VlvSTS* gene. For further study, tobacco plants were grown in the greenhouse, in containers filled with a sterilized soil mixture of peat and sand (1:1, *v*/*v*), at 22–24 °C, relative humidity of 60–70% and light intensity of 4000 lx. 

### 4.5. DNA Isolation from Plant Leaves

The genomic DNA from tobacco leaves was isolated using a Genomic DNA Purification Kit (Thermo Fisher Scientific, Vilnius, Lithuania). Leaf tissue, 50–100 mg, was crushed in a mortar with a pestle in the presence of liquid nitrogen. A sample was transferred to an Eppendorf microtube, and 200 µL of TE buffer and 400 µL of cell lysis buffer were added. The DNA was isolated in accordance with the manufacturer’s recommendations. After precipitation with ethanol, the DNA precipitate was dissolved in 50–100 µL of TE buffer, treated with RNase A, and then the resulting plant DNA was used as a template in PCR.

### 4.6. Polymerase Chain Reaction (PCR)

The PCR analysis of the *VlvSTS* and *npt*II genes was performed in 50 µL reaction mixture containing 0.5–1μg plant genomic DNA as the template, 50 mM KCl, 10 mM tris-HCl, pH 8.8 (at 25 °C), 1.5 mM MgCl2, 0.1% triton X-100, 0.2 mM dNTP mixture (Evrogen, Moscow, Russia), 50 pmol each *VlvSTS* and *npt*II primers, and 2.5 units Taq DNA polymerase (Thermo Fisher Scientific, Vilnius, Lithuania). As the *npt*II gene primers we used oligonucleotides specific to a 600-bp internal fragment of the gene: 5′-TATTCGGCTATGACTGGGCA-3′ and 5′-GCCAACGCTATGTCCTGATA-3′ [36]. The PCR was conducted under the following conditions: 5 min at 94 °C; 30 cycles: 1 min at 94 °C, 1 min at 58 °C, 2 min at 72 °C, followed by 7 min at 72 °C, on an MJ Mini Personal Thermal Cycler (Bio-Rad Laboratories, Singapore, Singapore). The PCR products were analysed by 0.9% agarose gel electrophoresis.

### 4.7. Semi-Quantitative RT-PCR and Quantitative Real-Time RT-PCR

The total RNA from tobacco leaves was isolated by the same method as the RNA from grape leaves [31]. The first cDNA chain was synthesized using an oligo(dT) primer and reverse transcriptase MMLV (Evrogen, Moscow, Russia) as recommended by the manufacturer. The semi-quantitative RT-PCR for tobacco RNA was performed under the same conditions as for the RNA from grape leaves and with the primers for the 3′-end of the *VlvSTS* gene (150 bp fragment length): 5′-TCAATTTAGAGAAAAAGAAACTCGAAGC-3′ and 5′-CAATCCAATCCTTCACCAGTGGTG-3′. The RT-PCR products were analyzed in 6% polyacrylamide gel. The mRNA content was determined by real-time PCR. The reaction was carried out in microtubes, in 25 µL of a mixture for real-time PCR, in the presence of EVAGreen (Syntol, Moscow, Russia), 0.4 µM of each primer, 1 µL of a cDNA sample, in a Rotor Gene 6000 Real-Time PCR Machine (Corbett Life Science, Mortlake, Australia), according to the following cycling profile: initial denaturation (95 °C, 5 min); 45 cycles: denaturation (95 °C, 15 s), annealing (56 °C, 20 s), elongation (72 °C, 20 s). For amplification, we used primers for the 3′-end of the *VlvSTS* gene (150 bp fragment length), as well as primers to the EF1-α gene (132 bp fragment length): 5′-TCCCACATTGCTGTCAAGTTTGC-3′ and 5′-GGGCTTGGTGGGAATCATCTTAAC-3′. Calculations were performed according to the 2^−ΔΔCT^ method [37], with EF1-alpha mRNA used as reference and the mRNA sample with the lowest transcription level (L24) as calibrator.

### 4.8. Biotests on Isolated Leaves of Transgenic Plants

The analysis of the transgenic plants resistance to pathogens was carried out on the leaves of 4–6 week old plants *in vitro*. *E. carotovora* was grown in LB liquid medium [29] overnight at 150 rpm and 28 °C. The bacterial culture was diluted to a density of 10^6^ cells/mL, and 7μLof the suspension was applied to the central vein of a leaf wounded with a needle. The leaves were placed on wet filter paper, and incubated at 24ºC under 16-h light day. The resistance of the leaves to *E. carotovora* was evaluated 48 h after infection. The area of leaf damage was scored 0 to 5 points, where 0 = no damage, 1 = 10–20% damage, 2 = 20–40%, 3 = 40–60%, 4 = 60–80%, 5 = 80–100%. The fungal strains were grown on glucose medium containing 2.0 g/L NH_4_NO_3_; 20.0 g/L glucose; 0.1 g/L MgSO_4_x7H_2_O; 3.0 g/L sucrose; 1.0 g/L KH_2_PO4; 1.0 g/L NaOH; 20.0 g/L agar in the dark for 3 to 4 days at 22–24 °C. Small pieces of mycelium of *Fusarium* or *B. cinerea* fungi were placed on the area of the central vein of leaves, placed on petri dishes with a wet filter. The degree of leaves damage by fungal pathogens was assessed 7–14 days after infection.

### 4.9. Obtaining Homozygous Lines of Transgenic Plants

In the inserted genetic construct, the target *VlvSTS* gene is linked to the *npt*II marker gene. Therefore homozygous transgenic tobacco lines were obtained using self-pollination and testing the seed resistance to kanamycin sulfate. Seeds of T0 transgenic plants were planted on MS medium with kanamycin sulfate (100 mg/L). Lines with a 3:1 segregation of kanamycin resistance were selected. These plants were transferred into the greenhouse to obtain seeds. T1 plants with 100% kanamycin-resistant seeds belonged to homozygous lines. We selected three homozygous lines from L10 line plants (L10-6, L10-8, L10-9) and two homozygous lines from L23 (L23-5, L23-9). The obtained lines were used in seed productivity tests.

### 4.10. Sample Preparation for Trans-Resveratrol Quantitative Determination

Leaves were obtained from control and transgenic plants grown for 2 months in the greenhouse. Leaf samples of 5 g were ground in liquid nitrogen and extracted with 25 mL of 80% methanol for 10–15min in an ultrasonic bath and then left overnight at 4 °C in the dark. The obtained extracts were centrifuged at 2000× *g* for 40 min; the supernatant was transferred to glass flasks and evaporated on a rotary evaporator at 40 °C. The residue was dissolved in 2 mL of 80% methanol and filtered through a 0.22 μm pore size PVDF filter (Biomed Scientific Ltd., Shenzhen, China). The obtained solutions were passed through Diapak C18 concentrating cartridges (ZAO BioKhimMak ST, Moscow, Russia) and the columns were washed with 2 mL of 80% methanol. The samples were stored in the dark at −20 °C.

### 4.11. High Performance Liquid Chromatography–Mass Spectrometry Determination of Trans-Resveratrol in Leaves of Transgenic Plants

Trans-resveratrol content in leaves of transgenic plants was determined using a Dionex Ultimate-3000 HPLC system (Thermo Fisher Scientific, San Jose, CA, USA) and a high-resolution Orbitrap Elite hybrid mass spectrometer (Thermo Fisher Scientific, Bremen, Germany). LC was performed on a Kinetex^®^ C18 phase column (100 × 2.1 mm, 1.7 μm sorbent particle size, 100 Å pore size) with solvents A (0.1% formic acid in deionized water) and B (0.1% formic acid in a mixture of 98% acetonitrile and 2% deionized water). The column was equilibrated with a mixture of 85% A and 15% B for 10 min at a flow rate of 250 µL/min. After sample injection onto the column, elution was performed under the following conditions: 5-min isocratic elution with 15% B, 5-min gradient increase from 5% to 80% B, 1-min gradient increase from 80 to 100% B, and 2-min isocratic elution with 100% B. Detection was performed on a mass-spectrometer in the selected ion monitoring (SIM) mode. Ions were recorded in a range of m/z values 229 ± 2.5. The capillary voltage was 3 kV. The measurement resolution was 240,000. The accuracy of mass determination was at least 5 ppm. Trans-resveratrol solution (Sigma-Aldrich, Saint-Louis, MO, USA) in 80% methanol was used as reference.

### 4.12. Total Anthocyanin Content in the Corollas of Tobacco Flowers 

Anthocyanins in flower corollas were determined by spectrophotometry. A weighed sample of the pigmented parts of corollas, 200–300 mg, was frozen in liquid nitrogen and ground in a mortar. Anthocyanin extraction and content calculation were performed as described in [38].

### 4.13. Quantitative Determination of Flavonoids in Plant Leaves and Flower Corollas 

Flavonoids were analyzed by differential spectrophotometry [39]. A weighed sample of the pigmented parts of corollas (200 mg) or leaves (1000 mg) was frozen and ground under liquid nitrogen. The extraction was carried out in a 95% ethanol solution with the addition of 0.01% Tween 80, by holding it twice at 50 °C for 20 min. We took 100–200 µL of the resultant extract, added 200 µL of a 5% alcohol solution of aluminum chloride, 20 µL of 0.1 N HCl and adjusted the volume to 2.5 mL. After 30 min, we measured the optical density at 412 nm. A rutin solution was used as reference.

### 4.14. Analysis of pollen VIABILITY, Pollen Grain Size and Quantity

Pollen fertility was estimated as follows. Flowers were collected before blossoming, at the stage of colored buds, and fixated with an ethanol-acetic acid mixture, 1:3. For fertility analysis, one flower was selected at random, its anthers were extracted, stained with acetocarmine, and a pressed specimen was prepared. At least 10 fields of view were examined. To determine the size and amount of pollen grains, a random flower was taken, anthers were extracted, macerated at 60 °C for 5 min in 2N HCl, and then the volume was brought in a measuring tube with distilled water to 2 mL. The resulting suspension was put into a counting chamber to count the grains: the amount of pollen grains in the flower was calculated by a formula. Pollen grains were counted in at least 10 chambers, in 20 large squares. The remaining macerate was used to determine the pollen grain volume. At least 15–20 random grains in 10 fields of view were taken into account. The volume of a pollen grain was determined based on the assumption that it had a spherical shape. The volume was calculated from the area and perimeter of a pollen grain projection using the Siams MesoPlant image analysis system (SIAMS, Ekaterinburg, Russia). The significance of differences in pollen fertility rate was determined using Fisher’s test, and that of differences in the number and volume of pollen grains, using the nonparametric Mann-Whitney test.

### 4.15. Statistical Analysis

The data were statistically analyzed using Statistica 6.0 software. Measurements were carried out in three biological and three analytical replicates. The diagrams and table show mean values and their standard deviations. The significance of differences was assessed based on the nonparametric Mann-Whitney test.

## 5. Conclusions

The study showed that the expression of a *VlvSTS* stilbene syntase gene in tobacco transgenic plants increases their resistance to bacterial pathogen *E. carotovora*. There was a significant reduction of disease symptoms after infection of leaves by grey mould fungus *B. cinerea*, but not to *Fusarium* fungi. We were the first to show that transgenic tobacco plants carrying the *VlvSTS* gene had a significantly larger pollen grain size and a smaller number of pollen grains per anther. The number of fertile pollen grains decreased, especially in the plant line with the highest expression of the *VlvSTS* gene. These changes resulted in a decreased weight of seed bolls in the transgenic tobacco lines.

## Figures and Tables

**Table 1:** Quantitative characteristics of pollen grains in tobacco plants with *VlvSTS* gene.

Plant Lines	Pollen Grain Volume, ×10^3^ µm^3^	Number of Pollen Grains per Flower, ×10^3^	Fertility, %
Control	8.49 ± 0.38	751.88 ± 98.5	85.62
L10	14.48 ± 0.81 **	485.00 ± 52.00 *	64.66 **
L21	12.14 ± 0.27 **	507.50 ± 61.45 *	79.45 *
L23	14.18 ± 0.72 **	691.25 ± 81.61	88.79
L24	10.17 ± 0.45 **	635.00 ± 66.58	87.44

* Statistically significant at Mann-Whitney test (*p* < 0.05).** Statistically significant at Mann-Whitney test (*p* < 0.01).

## Data Availability

Not applicable.
